# Antimicrobial Drugs in the Home, United Kingdom

**DOI:** 10.3201/eid1210.051471

**Published:** 2006-10

**Authors:** Cliodna A.M. McNulty, Paul Boyle, Tom Nichols, Douglas P. Clappison, Peter Davey

**Affiliations:** * Health Protection Agency, Gloucester, United Kingdom;; † University of Saint Andrews, Saint Andrews, United Kingdom;; ‡ Health Protection Agency, London, United Kingdom;; § Southwold Surgery, Southwold, United Kingdom;; ¶ University of Dundee, Dundee, United Kingdom

**Keywords:** Community, antibiotics, antimicrobial drugs, education, household survey, resistance, prescriptions, attitudes, antifungal drugs, research

## Abstract

A total of 6% of 6,983 households in the United Kingdom had leftover antimicrobial drugs, and 4% had standby antimicrobial drugs. Respondents with leftover drugs were more educated, more knowledgeable about antimicrobial drugs, younger, and female. Of respondents with leftover drugs, 44% kept them in case of future need, and 18% had taken these drugs without medical advice.

## Abstract 2

Persons more knowledgeable about these drugs are more likely to keep them.

Antimicrobial drug resistance is increasing worldwide and is related to use of these drugs (*1**,**2*). Use of leftover drugs may increase antimicrobial drug resistance in the community by exerting selective pressure in the commensal flora (*3**,**4*). Retention and use of leftover antimicrobial drugs are widespread in countries that sell antimicrobial drugs without a prescription (i.e., over-the-counter) (*5**,**6*). In countries in which over-the-counter antimicrobial drug sales are restricted, studies on use of leftover drugs have been few and mostly questionnaire-based, relying on respondent recall (*7**,**8*). A large survey of British household drug practices showed that 2% of medicines were leftover or standby drugs (*9*), but no information was obtained about the households containing them. To inform antimicrobial drug education campaigns (*10**,**11*) and research examining the relationship between antimicrobial drug prescribing and resistance (*12*), we conducted a household survey to identify antimicrobial drugs present and the characteristics of respondents who kept them.

## Methods

The survey formed part of the Office for National Statistics' Omnibus Survey in the United Kingdom in February, March, June, and July 2003. A representative sample of households was selected by using previously published methods (*9*). Households were invited by letter to participate in a study about their views and experiences on a variety of subjects. For households with >1 adult member, 1 person >16 years of age was selected by a random number tables. At the household visit, respondents were informed that the survey was sponsored by the department of health and was about drugs for treating infections. Respondents were asked to show the interviewer all drugs in the household that had been prescribed for infections, even if the person for whom they were prescribed was not present (temporarily or permanently), and asked whether the drugs were currently being used for an infection for which they were prescribed, had been prescribed for a previous infection (leftover drugs), or were being kept for use in the future (standby drugs). The amount of unused drug was noted and prescription details were recorded and coded by their listing in the British National Formulary. Antimicrobial drugs included all antifungal and antibacterial drugs.

Respondents were asked a series of questions about their use of antimicrobial drugs and whether they agreed or disagreed with a series of statements from education campaigns (*10**,**11*) about the usefulness of these drugs for coughs and colds, viruses, bacteria, and normal flora; the importance of completing courses of drug therapy, and antimicrobial drug resistance. Ethical approval was not required for this ongoing national survey, but respondents were able to refuse participation in any part of the survey at any time and were told that all information was anonymous and strictly confidential.

To enable comparison with nationally prescribed antimicrobial drugs, the number of community-prescribed drugs in England was obtained from prescribing analysis and cost data (PACT) (*13*). Sampling weights based on the 2001 census data for Great Britain were applied to household and respondent data to allow for any oversampling or undersampling by region, Carstair's deprivation quintile, age group, and sex. Multivariable logistic regression (ignoring sampling weights) was used to investigate independent associations between respondent characteristics and the presence of leftover antimicrobial drugs prescribed for the respondent.

## Results

**Figure 1:**
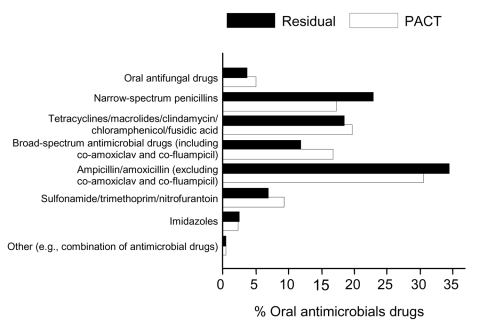
Oral leftover antimicrobial drugs compared with prescriptions issued (p = 0.16), United Kingdom, 2003. PACT, prescribing analysis and cost data.

Of 10,981 selected households, 25% refused to participate and 10% could not be contacted. Respondents in 7,120 (65%) households completed the questionnaire and 6,983 (64%) agreed to the drug survey. A total of 19% of the 6,983 households had an antimicrobial drug leftover (6%), kept for standby use (4%), or currently in use (11%) (Table 1). Six percent of leftover drugs had been prescribed for <3 days, 16% for 4–5 days, 61% for >6 days, and 17% were to be taken as required. No specific class of oral antimicrobial drugs was kept more often as leftover drugs relative to PACT prescription rates (Figure 1).

**Table 1:** Outcomes from household survey of antimicrobial drugs and interviews of an adult member of the household about behavior in relation to these drugs, United Kingdom, 2003*

Data		Antibacterial or antifungal drugs, % (95% CI)	Antibacterial drugs, % (95% CI)	Antifungal drugs, % (95% CI)
Household-based (6,983 households)				
	Households with drug	18.9 (18.0–19.9)	13.9 (13.1–14.8)	6.9 (6.3–7.6)
	Households with current drug	10.6 (9.9–11.4)	7.9 (7.2–8.5)	3.6 (3.2–4.1)
	Households with leftover drug	6.2 (5.7–6.8)	4.5 (4.0–5.0)	2.1 (1.8–2.5)
	Households with standby drug	3.7 (3.3–4.2)	2.4 (2.1–2.8)	1.5 (1.2–1.8)
	Households with leftover or standby drug	9.6 (8.9–10.3)	6.7 (6.1–7.3)	3.5 (3.1–4.0)
Antimicrobial drug–based (506 leftover, 292 standby)				
	Leftover drug >1 y old	38 (33–44)	32 (27–38)	55 (46–63)
	Standby drug >1 y old	31 (26–37)	29 (23–37)	36 (28–46)
	Leftover drug is more than half left	48 (43–53)	50 (44–56)	44 (36–53)
Respondent-based (7,120 respondents)				
	Respondent had a leftover drug that was originally prescribed	3.9 (3.4–4.4)†	2.8 (2.4–3.3)	1.2 (1.0–1.5)
	Respondent prescribed a drug in the last year	37.6 (36.4–38.8)		
	Respondent kept leftover drug from their most recent prescription	5.9 (5.0–7.0)		
	Respondent kept drug in case of future need	44 (35–52)		

*Some prescriptions contained both antibacterial and antifungal drugs and contribute to all 3 columns. CI, confidence interval. †This outcome is examined in more detail in Table 2.

**Figure 2:**
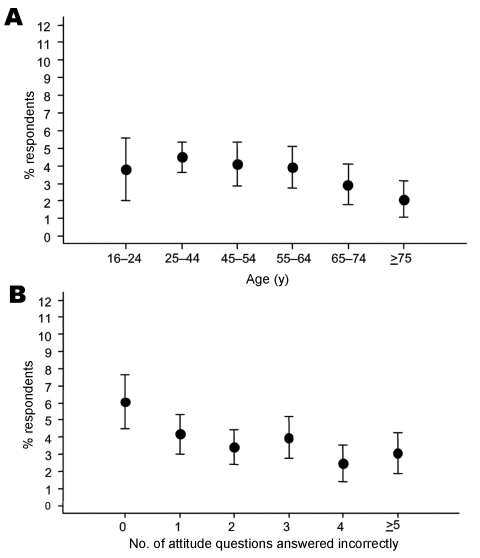
Percentage of respondents with a leftover antimicrobial drug (all antimicrobial drugs, i.e., antibacterial plus antifungal drugs) by A) age (y) of respondent (p = 0.01, by test for trend) and B) no. of attitude questions answered incorrectly (p = 0.002, by test for trend). Error bars show 95% confidence intervals.

Four percent of respondents had leftover antimicrobial drugs that had been prescribed. The most educated respondents were twice as likely as those least educated to have leftover antimicrobial drugs (5.4% with a university degree vs. 2.4% with no formal degree) (Table 2). Respondents most knowledgeable about antimicrobial drugs (6.1% if all 11 questions were answered correctly) were twice as likely as those least knowledgeable (3.1% if >5 were answered incorrectly) to keep them (Table 2, Figure 2). Level of education and knowledge of antimicrobial drugs were independently associated with being more likely to have leftover drugs (Table 2). Younger respondents and women were more likely to have leftover drugs (Table 2, Figure 2). Thirty-eight percent of respondents' leftover drugs had been dispensed >1 year earlier, and 48% of these drugs had more than half of the original amount remaining (Table 1). Of those who had kept a leftover antimicrobial drug prescribed in the past year, 44% did so for future need (Table 1). Of those with a leftover drug, 18% had taken antimicrobial drugs without advice compared with 4% of respondents who did not have a leftover drug (p<0.0005).

**Table 2:** Characteristics of respondents who had a leftover antimicrobial drug, United Kingdom, 2003*

Outcome: respondent had leftover drug originally prescribed for respondent				% (95% CI)			Crude OR† (95% CI)	p value	Adjusted OR† (95% CI)	p value
Age group, y										
		16–24		3.8 (2.4–6.0)			0.89 (0.82–0.98)	0.01	0.97 (0.88–1.06)	0.49
		25–44		4.5 (3.7–5.4)						
		45–54		4.1 (3.0–5.5)						
		55–64		3.9 (2.9–5.3)						
		65–74		2.9 (2.0–4.3)						
		>75		2.1 (1.3–3.5)						
Sex										
		Male		3.2 (2.6–4.0)			1		1	
		Female		4.4 (3.8–5.2)			1.38 (1.05–1.82)	0.02	1.22 (0.94–1.57)	0.14
Education										
		Degree		5.4 (4.1–7.1)			1	<0.0005	1	0.001
		Other		4.3 (3.6–5.1)			0.78 (0.56–1.10)		0.74 (0.54–1.02)	
		No formal degree		2.4 (1.8–3.2)			0.43 (0.29–0.65)		0.44 (0.29–0.68)	
No. questions regarding drugs answered incorrectly										
		0		6.1 (4.7–7.8)			0.87 (0.79–0.95)	0.002	0.91 (0.84-0.98)	0.02
		1		4.2 (3.1–5.5)						
		2		3.4 (2.6–4.5)						
		3		4.0 (2.9–5.4)						
		4		2.5 (1.6–3.8)						
		>5		3.1 (2.1–4.5)						
Carstair deprivation score										
	1st quintile (least deprived)				4.4 (3.4–5.9)	1.00 (0.91–1.11)		0.99	1.01 (0.92–1.10)	0.89
	2nd quintile				3.9 (2.8–5.5)					
	3rd quintile				3.4 (2.6–4.6)					
	4th quintile				3.1 (2.3–4.1)					
	5th quintile (most deprived)				4.6 (3.6–5.8)					
Prescribed a drug in the past year										
			No		2.1 (1.7–2.6)	1			1	
			Yes		6.9 (5.9–8.0)	3.51 (2.66–4.65)		<0.0005	3.50 (2.69–4.54)	<0.0005

*CI, confidence interval; OR, odds ratio. †For age group, no. of questions regarding drugs answered incorrectly used Carstair deprivation score; the odds ratio is between adjacent categories. The corresponding p value is a test for trend.

When directly questioned, 38% of respondents (95% confidence interval 36.4–38.8%) recalled that they had been prescribed a drug in the past year. Younger age in women (50% if 16–24 years of age, 43% if 25–44 years of age, and 44% if 45–54 years of age) and lower level of education (33% with a degree and 40% with no formal qualifications on leaving school at the minimum age [16 years]) were independently associated with being prescribed an antimicrobial drug.

## Discussion

This large survey of household antimicrobial drugs, which was conducted in a country with restricted over-the-counter sales, showed an association between keeping leftover antimicrobial drugs and higher education and knowledge about these drugs, younger age, and being female. The strength of this study is that we asked a large representative sample to show us their antimicrobial drugs rather than relying on respondent memory. However, we may not have been shown all antimicrobial drugs, and some of the leftover drugs may have been misclassified as standby drugs. The characteristics of respondents in households with standby drugs were similar to those of respondents with leftover drugs. The number of antimicrobial drugs found was twice that found by a recent survey in Sweden that relied on respondent recall; in that study, the respondents also reported a lower number of antimicrobial drug prescriptions (*8*).

Persons may keep leftover antimicrobial drugs because too much was prescribed for their initial infection. Most community-acquired infections are respiratory or urinary, for which many prescribed courses of antimicrobial drugs are longer than necessary (*14*). If the standard duration of treatment could be shortened and package size reduced to contain enough drug for 3 to 5 days, the temptation to keep antimicrobial drugs might be diminished. Our finding supports this suggestion because prescriptions for >6 days constituted 61% of leftover drugs, whereas prescriptions for <3 days constituted 6% of leftover drugs. Evidence shows that repeated treatment with antimicrobial drugs exerts greater selective pressure on normal bacterial flora than a single course of treatment (*14**,**15*). Consequently, persons who use leftover antimicrobial drugs repeatedly are at greater risk for colonization and infection with drug-resistant organisms (*1**,**3**,**4**,**15*).

Our results show that any public education campaign to reduce retention and use of leftover antimicrobial drugs must be directed at well-educated groups rather than the less educated, who are prescribed more antimicrobial drugs. One explanation may be that well-educated people are confident that they can make their own decisions about antimicrobial drug use, and this may be particularly relevant when their infection is less severe or becomes asymptomatic. Furthermore, they do not discard these drugs because 44% of those with leftover antimicrobial drugs admitted keeping them in case of future need. Antimicrobial drug education campaigns should reinforce the message that leftover drugs should be returned to the pharmacist or that these drugs should only be taken after the advice of a health professional.

Our finding of high levels of leftover antimicrobial drugs suggests that prescription does not equate to use. This will be an important source of bias in epidemiologic studies examining risk factors for antimicrobial drug resistance (*12*) that include dose and duration of antimicrobial drug treatment in their analysis of exposure (*1**,**4*).
